# Relaxation time approximations in PAOFLOW 2.0

**DOI:** 10.1038/s41598-022-08931-5

**Published:** 2022-03-23

**Authors:** Anooja Jayaraj, Ilaria Siloi, Marco Fornari, Marco Buongiorno Nardelli

**Affiliations:** 1 Department of Physics, University of North Texas, Denton, TX 76203 USA; 2 Department of Physics and Astronomy, University of Southern California, Los Angeles, CA 90007 USA; 3 Department of Physics and Science of Advanced Materials Program, Central Michigan University, Mt. Pleasant, MI 48859 USA; 4 Santa Fe Institute, Santa Fe, NM 87501 USA

**Keywords:** Condensed-matter physics, Theory and computation, Electronic structure, Thermoelectrics

## Abstract

Regardless of its success, the constant relaxation time approximation has limited validity. Temperature and energy dependent effects are important to match experimental trends even in simple situations. We present the implementation of relaxation time approximation models in the calculation of Boltzmann transport in PAOFLOW 2.0 and apply those to model band-structures. In addition, using a self-consistent fitting of the model parameters to experimental conductivity data, we provide a flexible tool to extract scattering rates with high accuracy. We illustrate the approximations using simple models and then apply the method to GaAs, Si, $\hbox {Mg}_3\hbox {Sb}_2$, and $\hbox {CoSb}_3$.

## Introduction

Over the years, first-principles calculations have become a complementary tool for the experimental research aiming to discover and understand materials with relevant electronic properties. This has greatly improved also the understanding of the electronic transport properties which are crucial in applications ranging from electronics to energy conversion. With electronic transport coefficients, we indicate the response function to an applied external electric field and/or temperature gradient. The quantities of interest are the electric conductivity, $\sigma$, the electronic therma conductivity, $\kappa _e$, and the Seebeck coefficient, *S*, which couples the heat and charge fluxes. These quantities are, in the linear regime, constant tensors and include dissipation phenomena.

The most standard approach in calculating the transport coefficient is to use the semi-classical Boltzmann theory within the constant relaxation time approximation (CRTA). In most cases, this has been used without much analysis. Though it works for certain systems, recent research has shown that the CRTA have resulted in wrong predictions, missing vital information to understand the transport properties^1–3^. Moreover, the introduction of an arbitrary constant relaxation time severely limits the predictive capabilities of first-principles band-structures. In this work, we investigate how the CRTA affects the electric conductivity of well known materials by considering different scattering models that include energy and temperature dependence. Though methods exist for ab initio calculation of electron–phonon relaxation times^4,5^, the calculation of the electron–phonon matrix requires extremely dense ${\mathbf{k}}$ and ${\mathbf{q}}$ point meshes. Therefore, prohibitively high computational costs make these techniques of limited practicality, especially when aiming to data driven high-throughput approaches. We chose to combine accurately interpolated band-structures and simplified mathematical models of the scattering phenomena in order to explore the consequences on the transport coefficients beyond CRTA and parabolic bands. Extrinsic scattering mechanisms (impurities, grain boundaries, alloy disorder) contribute significantly to the transport properties in a system. Indeed, these extrinsic scattering mechanisms may be often tuned during the synthesis of the system^6^ and often beyond the power of predictive computational methods. The contribution of intrinsic scattering mechanism in a system, on the other hand, can be more easily addressed to obtain optimized performances. In addition to the insight offered by relaxation time and transport data studies offered by other methods^7,27^, in this work, we introduce a self-consistent fitting of transport properties to experimental data which will give us an understanding of the temperature dependence of various scattering mechanisms for specific experimental samples. These relaxation time approximation (RTA) models are coded in our recently released PAOFLOW package^8,9^. In this paper, we will discuss in detail the theory and implementation of the RTA models in the newest release of the software, and illustrate the automated workflow while, at the same time, documenting the influence of scattering phenomena in the band-structure of cubium, graphene, and selected materials: Si, GaAs, Mg$_3$Sb$_2$, and CoSb$_3$.

## Methods

### PAOFLOW

PAOFLOW is a software tool to efficiently post-process standard first-principles electronic structure plane-wave pseudopotential calculations in order to promptly compute, from interpolated band-structures and density of states, several quantities that provide insight on transport, optical, magnetic and topological properties such as anomalous and spin Hall conductivity, magnetic circular dichroism, spin circular dichroism, and topological invariants. The methodology is based on the projection on pseudo-atomic orbitals (PAO) discussed in detail in Refs.^10–12^.

Accurate PAO Hamiltonian matrices can be built from the direct projection of the Kohn–Sham (KS) Bloch states $| \psi _{n{{\mathbf {k}}}} \rangle$ onto a chosen basis set of fixed localized functions. The Hamiltonian for a specific material, $\hat{H}\left( {\mathbf{R}}\right)$, is computed in real space using atomic orbitals or pseudo atomic orbitals from the pseudopotential of any given element. The key, in this procedure, is in the mapping of the ab initio electronic structure (solved on a well converged and large plane waves basis set) into tight-binding (TB) formalism that precisely reproduces a selected number of bands of interest. The crucial quantities that measure the accuracy of the basis set are the projectabilities $p_{n{{\mathbf {k}}}}=\langle \psi _{n{\mathbf {k}}} | \hat{P} | \psi _{n{\mathbf {k}}} \rangle \ge 0$ ($\hat{P}$ is the operator that projects onto the space of the PAO basis set, as defined in Ref.^11^) which indicate the representability of a Bloch state $| \psi _{n{\mathbf {k}}} \rangle$ on the chosen PAO set. Maximum projectability, $p_{n{\mathbf {k}}}= 1$, indicates that the particular Bloch state can be perfectly represented in the chosen PAO set; contrarily, $p_{n{\mathbf {k}}} \approx 0$ indicates that the PAO set is insufficient and should be augmented. Once the Bloch states with good projectabilities have been identified, the PAO Hamiltonian is constructed as

1 $$\begin{aligned} \hat{H}({\mathbf {k}}) = AEA^\dagger + \chi \left( I-A \left( A^{\dagger }A \right) ^{-1}A^\dagger \right) . \end{aligned}$$

Here *E* is the diagonal matrix of KS eigenenergies and *A* is the matrix of coefficients obtained by projecting the Bloch wavefunctions onto the PAO set. Since the filtering procedure introduces a null space, the parameter $\chi$ is used to shift all the unphysical solutions outside a given energy range of interest. The procedure in Eq. (1) is recommended for most cases.

Band-structure interpolation on arbitrary Monkhorst and Pack (MP) **k**-meshes for the integration in the Brillouin zone (BZ) are at the very core of the ability of PAOFLOW to provide high-precision electronic structure data. Indeed, the TB Hamiltonian can be Fourier transformed from real space representation to the **k**-space and interpolated using an efficient procedure based on a zero-padding algorithm and fast Fourier transform routines.

The same accuracy defined by the projectabilities is conserved in this process. The expectation values of the momentum operator, which is the main quantity in the definition of the transport coefficients, is given by

2 $$\begin{aligned} {\mathbf{p}}_{nm} ({\mathbf{k}})= & {} \left\langle \psi _n ({\mathbf{k}}) |\hat{p} | \psi _m ({\mathbf{k}}) \right\rangle \nonumber \\= & {} \left\langle u_n ({{\mathbf{k}}}) |\frac{m_0}{\hbar } {\varvec {\nabla }}_{{\mathbf{k}}} \hat{H}({\mathbf{k}})| u_m ({\mathbf{k}}) \right\rangle \end{aligned}$$

with

3 $$\begin{aligned} {\varvec {\nabla }}_{\mathbf{k}} \hat{H}({\mathbf{k}}) = \sum _{\mathbf{R}} i{\mathbf{R}} \exp \left( i{\mathbf{k}}\cdot {\mathbf{R}}\right) \hat{H}\left( {\mathbf{R}}\right) . \end{aligned}$$

$\hat{H}\left( {\mathbf{R}}\right)$ being the real space PAO matrix and $| \psi _n ({\mathbf{k}}) \rangle = \exp (-i {\mathbf{k}} \cdot {\mathbf{r}}) | u_n ({\mathbf{k}}) \rangle$ the Bloch’s functions^13^.

### Boltzmann transport

In PAOFLOW the electrical conductivity is evaluated by solving the semi-classical Boltzmann equation (BTE) that describes the evolution of the distribution function *f* of an electron gas under external electric field and in presence of scattering mechanisms^14–16^. In the scattering-time approximation, the conductivity tensor $\sigma _{ij}$ can be expressed as an integral over the first BZ:

4 $$\begin{aligned} \sigma _{ij}= \frac{e^2}{4\pi ^3} \int _{BZ} \sum _n \tau _n({\mathbf{k}}) v_n^i({\mathbf{k}})v_n^j({\mathbf{k}}) \left( -\frac{\partial f_0}{\partial E}\right) d {\mathbf{k}}, \end{aligned}$$

where $\tau _n({\mathbf{k}})$ is the relaxation-time, $v_n^i({\mathbf{k}})$ is the *i*-th component of the electron velocity corresponding to the *n*-th band for each **k**-point in the BZ (${\mathbf{v}}_n$ is derived by the diagonal of the momentum matrix element, Eq. (2)), $f_0$ is the equilibrium distribution function, and *E* is the electron energy.

Generalizing Eq. (4) it is also possible to define analogue expressions for the Seebeck coefficient *S* and the electron contribution to thermal conductivity $\kappa _{el}$. Following the notation of Ref.^17^, we introduce the generating tensors ${\mathscr {L}}_{\alpha }$ ($\alpha = 0, 1, 2$):

5 $$\begin{aligned} {\mathscr {L}}_{\alpha } = \frac{1}{4\pi ^3} \int \sum _n \tau _n({\mathbf{k}}) {\mathbf{v}}_n({\mathbf{k}}){\mathbf{v}}_n({\mathbf{k}}) \left( -\frac{\partial f_0}{\partial E}\right) \left[ \epsilon _n({\mathbf{k}})-\mu \right] ^{\alpha }d {\mathbf{k}}, \end{aligned}$$

where ${\mathbf{v}}_n({\mathbf{k}}){\mathbf{v}}_n({\mathbf{k}})$ indicates the dyadic product, $\epsilon _n({\mathbf{k}})$ the band-structure, and $\mu$ is the chemical potential. The coefficients $\sigma$, *S* and $\kappa _{el}$ can be expressed as follows:

6 $$\begin{aligned} \sigma= & {} e^2{{\mathscr {L}}}_0, \nonumber \\ S= & {} -\frac{1}{T e} \left[ {\mathscr {L}}_0\right] ^{-1} \cdot {\mathscr {L}}_1, \nonumber \\ \kappa _{el}= & {} \frac{1}{T} \left( {\mathscr {L}}_2 - {\mathscr {L}}_1 \cdot \left[ {\mathscr {L}}_0\right] ^{-1} \cdot {\mathscr {L}}_1\right) , \end{aligned}$$

where *T* is the temperature. Our formalism based on PAO-TB performs the computations of the band-velocities and avoids issues with possible band-crossing. In addition, from Eqs. (4–5), it is evident that the evaluation of the transport properties requires an accurate integration over a fine grid of k-point in the BZ which becomes a trivial task using the TB representation from the PAO projections and Eq. (3).

### Relaxation time approximation

The most common implementations of the Boltzmann transport equations assume the scattering time $\tau$ to be a constant (CRTA). A constant $\tau$ factors out of Eq. (5) and, thus, the method returns the quantities $\sigma _0 = \sigma /\tau$ and $\kappa _{el,0} = \kappa _{el}/\tau$ (in the Seebeck coefficient $\tau$ cancels out). Clearly this is a severe approximation for a quantity that is expected to be significantly temperature-dependent and energy-dependent (typically via a power law). A direct estimate of the dependence of $\tau$ on energy and temperature is an important complement to any transport study, and would provide, even if at a phenomenological level, important insight into the relevant scattering mechanism present in any given system. Moreover, a direct comparison with existing experimental data would provide an extra layer of characterization for real world applications.

The approach implemented in PAOFLOW is based on the work of Jacoboni et al.^18^ and recently included in the BoltzTrap^19^ framework by the group of Fiorentini^20^. They employed analytical energy-dependent expressions for the relaxation time, which were developed on the basis of known semiclassical theories and include the most important mechanisms of electron scattering by acoustic phonons, polar-optical phonons, and charged impurities.

Acoustic phonon scattering is treated within the elastic deformation potential approach in the long-wavelength acoustic-phonon limit,

7 $$\begin{aligned} \tau _{ac}(E,T)= \frac{2\pi \hbar ^{4}\rho v^{2} }{(2m^{*})^{\frac{3}{2}}k_BT D_{ac}^{2} \sqrt{E}}, \end{aligned}$$

where *E* is the band structure energy considered from the top of the valence bands and T is the temperature. All other parameters are defined in Table 1.

Similarly to the assumptions that were used in acoustic phonon scattering, we model optical phonon scattering with an elastic deformation potential ($D_{op}$):

8 $$\begin{aligned}&\tau _{op}(E,T)= \frac{\sqrt{2k_{B}T}\pi x_{o}\hbar ^{2}\rho }{m^{{*}^{\frac{3}{2}}}D_{op}^{2}[\ N_{op}\sqrt{x+x_{o}}+(N_{op}+1)\Theta (x-x_{o})\sqrt{x-x_{o}}]\ }, \end{aligned}$$

9 $$\begin{aligned}&N_{op} = \frac{1}{\exp \frac{{\hbar \omega _{op}}}{k_B{T}}-1}, \nonumber \\&x = \frac{E}{k_{B}T}, \nonumber \\&x_{o} = \frac{\hbar \omega _{op}}{k_{B}T}. \end{aligned}$$

The first term in the denominator of Eq. (8,9) represents the absorption of optical phonons and the second term represents the emission of the optical phonons. The probability of emission of a phonon when $E < \hbar \omega _{op}$ is zero since the electron does not have enough energy to emit the phonon and this is represented by the Heaviside step function $\Theta$ included in the second term. $N_{op}$ represents the number of optical phonons. Polar optical scattering is modeled following Ridley:^21^

10 $$\begin{aligned} \tau _{pop}(E,T) = \sum _{i}\frac{Z(E,T,\omega _{i}^{l})E^{\frac{3}{2}}}{C(E,T,\omega _{i}^{l})-A(E,T,\omega _{i}^{l})-B(E,T,\omega _{i}^{l})} \end{aligned}$$

where the sum is over all longitudinal-optical phonons, with energy $\omega _{i}^{l}$; the functions *A*, *B*, *C*, and *Z* are omitted for brevity and can be found in Appendix I of the Supplementary Information.

Note that the deformation potential theory used to model the electron phonon interaction assumes that the atomic displacements can be described by long wavelength atomic and optical waves^18,37^. This is a wide spread assumption commonly used in semiconductors since carriers are typically confined to narrow energy windows around the band extrema (therefore the scattering mechanism will be dominated by long wavelength phonons). However, this approximation should be used with caution in systems where short wavelength phonon scattering is known to be important. For impurity scattering we use the Brooks–Herring approach:^22^

11 $$\begin{aligned} \tau _{imp}(E,T) = \frac{E^{\frac{3}{2}} \sqrt{2m^{*}}4\pi \varepsilon ^2}{\left( log\left( 1+\frac{1}{x}\right) -\frac{1}{1+x}\right) \pi n_I Z_I^{2}e^{4}} \quad {\text {with}} \quad x = \frac{E}{k_{B}T}. \end{aligned}$$

Finally, in semiconducting compound the strain induced by acoustic phonons creates a piezoelectric field. This piezoelectric scattering is modelled as in Ref.^18^.

12 $$\begin{aligned} \tau _{pac}(E,T) = \frac{\sqrt{2E}2\pi \varepsilon ^2\hbar ^2\rho v^2}{p^2 e^2 \sqrt{m^*} k_BT} \times \left[ 1-\frac{\epsilon _{o}}{2E}\log \left( 1+4\frac{E}{\epsilon _{o}}\right) +\frac{1}{1+4\frac{E}{\epsilon _{o}}}\right] \end{aligned}$$

where $\varepsilon =\epsilon _{o}+\epsilon _{\infty }$ and the piezoelectric effect is captured by the piezoelectric constant, *p*.

The global relaxation time is then obtained using Matthiessen’s rule:

13 $$\begin{aligned} \frac{1}{\tau _{total}(E,T)}=\frac{1}{\tau _{imp}(E,T)}+\frac{1}{\tau _{ac}(E,T)} +\frac{1}{\tau _{op}(E,T)}+\frac{1}{\tau _{pop}(E,T)}+\frac{1}{\tau _{pac}(E,T)}. \end{aligned}$$

**Table 1:** Symbols and units for the scattering parameters required in various scattering models.

Parameter	Symbol	Units
Mass density	$\rho$	kg/m$^{3}$
Lattice constant	a	m
Low freq. dielectric constant	$\epsilon _{0}$	–
High freq. dielectric constant	$\epsilon _{\infty }$	–
Acoustic velocity	v	m/s
Effective mass ratio	$m^{*}$	–
Acoustic deformation potential	$D_{ac}$	eV
Optical deformation potential	$D_{op}$	eV
Optical phonon energy	$\hbar \omega _{op}$	eV
Number of impurities	$n_{I}$	cm$^{-3}$
Charge on impurity	$Z_{I}$	–
Piezoelectric constant	p	C/m$^{2}$

## Simple models and the parabolic band approximation

In order to quantify the improvement of a richer RTA, and to gain a better understanding how varying various parameters affect the overall transport properties of a system, we start with two simple TB models: cubium and graphene. Cubium was chosen as representative of a 3D solid with quasi-parabolic bands (near the BZ center, $\Gamma )$ and graphene for its 2D character and its linear dispersion at the Fermi level. The TB Hamiltonian for a system with two atoms per unit cell with contributions from a single orbital is given by

$$\begin{aligned} {\mathscr {H}}({\mathbf{k}} ) = \begin{bmatrix} E_{g}/2 &{}\quad -t\Delta _{\mathbf{k }} \\ -t\Delta _{\mathbf{k} }^{*} &{}\quad -E_{g}/2 \\ \end{bmatrix}, \end{aligned}$$

where t is the first nearest-neighbor hopping parameter and $E_{g}$ defines the band gap of the band-structure. $\Delta _{\mathbf{k }} = \sum _{\varvec{\delta }}e^{i{\mathbf{k}} .\varvec{\delta }}$ and $\delta$ runs over nearest neighbors. For the cubium, because of its simple structure, the vectors for the six nearest neighbours are $\delta = a(\pm 1,0,0),a(0,\pm 1,0),a(0,0,\pm 1)$, where *a* is the lattice constant, so that:

$$\begin{aligned} \Delta _{\mathbf{k }}&= e^{ik_{x}a} + e^{-ik_{x}a} + e^{ik_{y}a} +e^{-ik_{y}a} + e^{ik_{z}a} + e^{-ik_{z}a}\\&= 2(\cos {k_{x}a}+\cos {k_{y}a}+\cos {k_{z}a}). \end{aligned}$$

Similarly for graphene, the vectors for the three nearest neighbors are $\delta = \frac{a}{2}(1,\sqrt{3})$, $\frac{a}{2}(1,-\sqrt{3}), -a(1,0)$ and

$$\begin{aligned} \Delta _{\mathbf{k }}&= e^{i{\mathbf{k}} .\varvec{\delta }_{1}} + e^{i{\mathbf{k}} .\varvec{\delta }_{2}} + e^{i{\mathbf{k}} .\varvec{\delta }_{3}} \\&= e^{-ik_{x}a}\left[ 1 + 2e^{3ik_{x}a/2}\cos {\frac{\sqrt{3}k_{y}a}{2}}\right] . \end{aligned}$$

Setting $E_{g}$ to 0 eV reproduces a graphene like band-structure where the bands show a linear dispersion at the Dirac point *K* in the BZ.

Since the energy and temperature dependence of the functional form of the scattering times were obtained using a parabolic band approximation, it is useful to examine the validity of such an approximation within electronic transport. The formulas for transport coefficients for a parabolic band within the CRTA, derived in detail in Ref.^17^, have been compiled in Eqs. (14)–(19).

**Figure 1:**
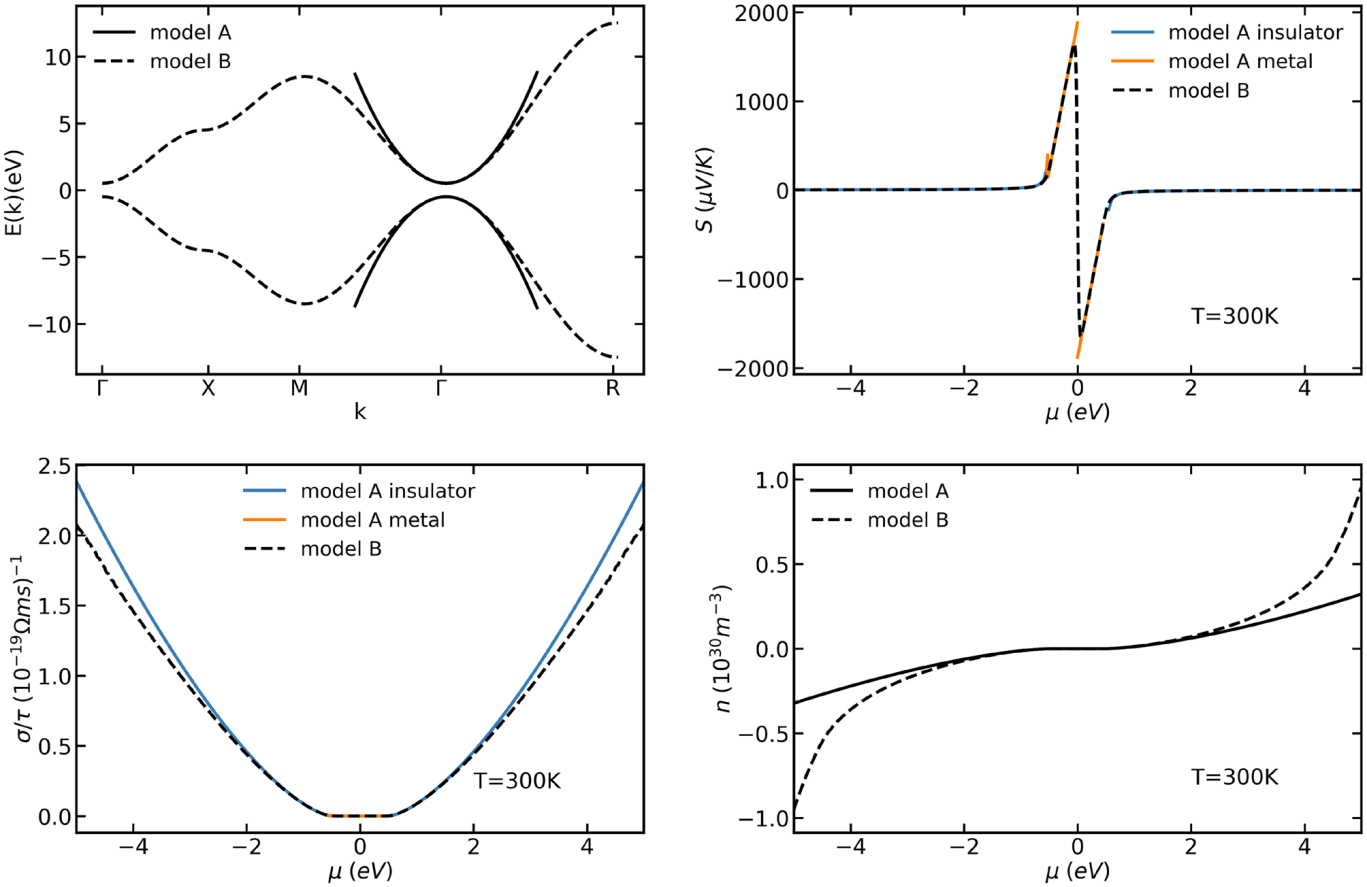
Band-structure of a cubium with two bands (top left panel, dashed line) and a parabolic fit near $\Gamma$ point (solid line). In the top right, bottom left, and bottom right panels, the Seebeck coefficient, the conductivity, and the carrier concentration, respectively, are reported.

In the insulating regime for parabolic bands, when $\beta (E_{n}-\mu )\gg 1$

14 $$\begin{aligned}&{[} \varvec{\sigma }]\ _{i,j} = \frac{e^{2}\tau 2^{3/2}\sqrt{m_{x}m_{y}m_{z}}}{3\pi ^2\hbar ^{3}m_{i}} [\ m_{n}(\mu -E_{n})]\ ^{3/2} \delta _{i,j} \end{aligned}$$

15 $$\begin{aligned}&{[} {\mathbf{S}} ]\ _{i,j} = -m_{n}\frac{k_{B}}{2e}\left[ \ 5+m_{n}2\beta (E_{n}-\mu )\right] \ \delta _{i,j} \end{aligned}$$

16 $$\begin{aligned}&n = \frac{\sqrt{m_{x}m_{y}m_{z}}{\exp (-\beta (E_{n}-\mu ))}}{\sqrt{2}\hbar ^{3}\pi ^{3/2}\beta ^{3/2}} \end{aligned}$$

and in the metallic regime for parabolic bands, when $\beta (E_{n}-\mu )\ll -1$

17 $$\begin{aligned}&{[}\ \varvec{\sigma }]\ _{i,j} = \frac{e^{2}\tau 2^{3/2}\sqrt{m_{x}m_{y}m_{z}}}{3\pi ^2\hbar ^{3}m_{i}}[\ m_{n}(\mu -E_{n})]\ ^{3/2} \delta _{i,j} \end{aligned}$$

18 $$\begin{aligned}&{[} {\mathbf{S}} ]\ _{i,j} = -\frac{k_{B}\pi ^{2}}{2e\beta (E_{n}-\mu )} \delta _{i,j} \end{aligned}$$

19 $$\begin{aligned}&n = {\left[ \ \frac{{-2(m_{x}m_{y}m_{z})^{1/3}}(E_{n}-\mu )}{3^{2/3}\hbar ^{2}\pi ^{4/3}}\right] \ }^{3/2} \end{aligned}$$

where, $E_{n}$ represents either a band-edge minimum or maximum, and $m_{n}$ is +1 for a conduction-like band, and -1 for a valence-like band, $m_{x}, m_{y}$ and $m_{z}$ are the x, y and z components of the effective mass, $\mu$ is the chemical potential of interest, $\beta =\frac{1}{k_{B}T}$ and *n* is the charge carrier concentration. We use the cubium model (Eq. 21) and compare the transport properties to those of the parabolic bands in Fig. 1:

20 $$\begin{aligned} & {\text {model A (Parabola)}:} E({\mathbf{k}} ) = -\hbar ^2|{\mathbf{k}} |^2/2m, \end{aligned}$$

21 $$\begin{aligned}&{\text {model B (Cubium)}:} E({\mathbf{k}} ) = -6+2(cos(k_{x}a)+cos(k_{y}a)+cos(k_{z}a)), \end{aligned}$$

22 $$\begin{aligned}{\text {model C (Graphene)}:} E({\mathbf{k}} ) = \pm 2.7\sqrt{1+4\cos \left( \frac{3}{2}k_{x}a\right) \cos \left( \frac{\sqrt{3}}{2}k_{y}a\right) +4\cos ^{2}\left( \frac{\sqrt{3}}{2}k_{y}a\right) } \pm 0.25. \end{aligned}$$

As apparent from the top left panel of Fig. 1, the cubium bands start deviating from the parabolic bands at $\sim$ 1.5 eV. Subsequently, all the transport properties for the parabolic model and the cubium band-structure are expected to match from 0 eV to $\sim$1.5 eV as the cubium band is a good approximation of a parabolic band in this range. This is reflected in the rest of the panels of Fig. 1. Since the parabolic band approximation is no longer valid in the cubium model after this limit, the transport properties of cubium start to deviate from those calculated using Eqs. (14)–(19). This deviation from parabolicity in transport properties is slightly enhanced at $\sim$4 eV due to contributions from the flat feature of the bands of cubium around the X point of the band-structure.

**Figure 2:**
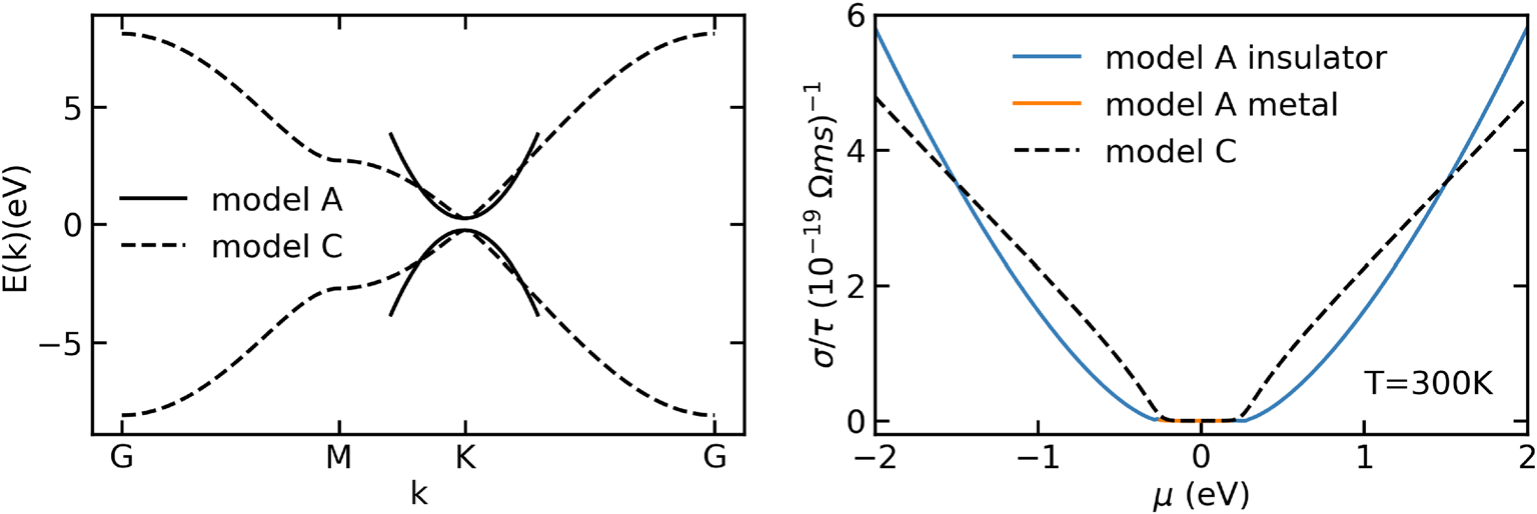
Band-structure of a graphene model and parabolic fit at *K* (left panel, dashed and solid line, respectively). The right panel shows the corresponding conductivities computed with Eqs. (14), (17), and PAOFLOW.

In Fig. 2, we consider a graphene like band-structure with a band gap of 0.5 eV. As expected, graphene bands do not follow a parabolic approximation, except very close to the Fermi surface. Therefore, the conductivity calculated using a parabolic band approximation is able to reproduce the conductivity calculated using BTE only extremely close to the valence and conduction band edges.

### Simple models beyond CRTA

Simple models allow to investigate the effect of improving the CRTA with minimal computational overload and monitor the consequences in physically transparent scenarios. Our simplified approach is conducive to an exploration of the parameters’ space of the RTA models which can be then used with more realistic band-structures. We can control the parameters of the models to enhance one or the other scattering mechanisms by varying the values of the deformation potentials, or of the relevant optical frequencies or of the sound velocity, to name a few. This can give insight into the potential design of materials with optimal properties for any given application.

**Figure 3:**
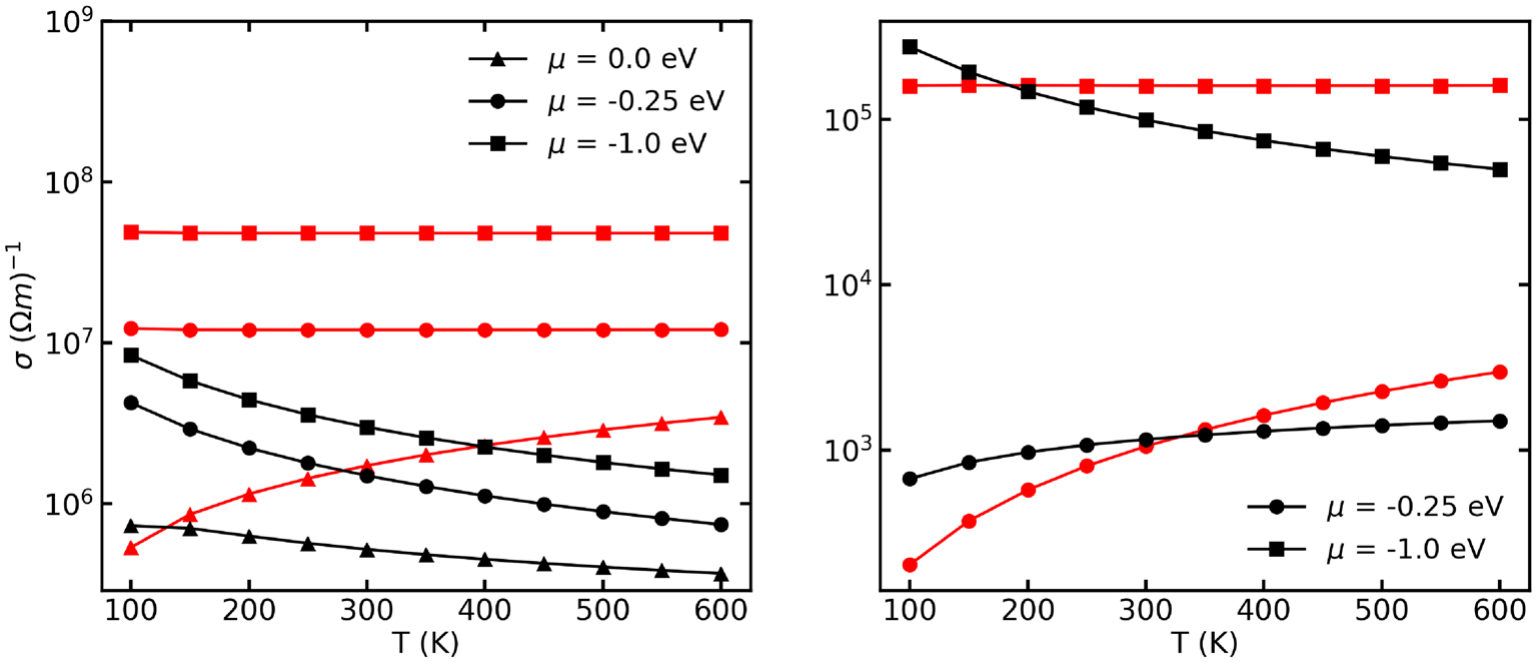
Electronic conductivity as a function of temperature at various chemical potentials (representing the doping level, referred to the middle of the gap). The left panel corresponds to graphene (with zero band gap) and the right panel corresponds to cubium with a band gap of 0.5 eV. The red lines denote the conductivity calculated using the CRTA whereas the black lines correspond to the conductivity calculated using the RTA; different markers correspond to different chemical potentials as in the legends.

A first observation involves the comparison between the conductivity calculated within CRTA and other other RTA models. We present results for graphene and cubium, Fig. 3. Experimentally, the distinction between semiconducting and metallic behavior (including the case of heavy doping) is understood in term of the temperature dependence of the conductivity. Samples whose conductivity increases with temperature are semiconducting and samples whose conductivity decreases with temperature are metallic. In semiconductors, the increase in the number of charge carriers prevails over the reduction of the relaxation time; in metals, the reduction of $\tau$ induces the reduction of $\sigma$.

In the case of graphene (a quasi-linear dispersion), the CRTA ($\tau = 10^{-14}$ s) provides temperature-independent conductivity in the metallic case and monotonically increasing conductivity in the semiconducting regime (the position of the chemical potential wrt the band edge determine the regime from the electronic structure point of view): only the variation of the carrier density due to temperature is captured in the calculation (Fig. 3, left panel, red lines). When applied to a two-band cubium with a forbidden energy gap of 0.5 eV, the same phenomenology is obtained in the CRTA (Fig. 3, right panel, red lines). Let’s consider a specific RTA model constructed using $[{D_{ac}:1,\rho :1e3,v:1e3,ms:1,D_{op}:5e10,h\omega _{lo}:0.01}]$ and optical phonon and acoustic phonon scattering mechanisms (see section “Relaxation time approximation”). In graphene, the chosen RTA model introduce dissipation phenomena that shorten $\tau$ as the temperature increases: this induce a reduction in conductivity. In the cubium model, we recover the experimental evidence of decreasing conductivity as function of temperature in the heavily-doped (metallic case) and increasing conductivity as function of temperature in the semiconducting regime.

## Relaxation time models and experimental conductivity

The scattering models described in section “Relaxation time approximation” have limited validity when extrinsic effects such as size of dopants or impurities, edge effects, etc. can significantly affect the scattering time. The electronic conductivity calculated using the relaxation times from the models, however, provide a framework for comparison to experiments and provide insight on the wide variations in experimental conditions and doping. We propose a modified Mathiessen’s rule as:

23 $$\begin{aligned} \frac{1}{\tau _{total}(E,T)}=\frac{a_{imp}(T)}{\tau _{imp}(E,T)}+\frac{a_{ac}(T)}{\tau _{ac}(E,T)} +\frac{a_{op}(T)}{\tau _{op}(E,T)}+\frac{a_{pop}(T)}{\tau _{pop}(E,T)}+\frac{a_{pac}(T)}{\tau _{pac}(E,T)}. \end{aligned}$$

In the above equation, $a_{imp}$, $a_{ac}$, $a_{op}$, $a_{pop}$ and $a_{pac}$ are correcting functions that are fitted to reproduce the experimental conductivity. The fitting procedure uses the sequential least squares programming (SLSQP)^23^ method, which allows for constrained non linear optimization of the fitting functions. A more detailed discussion of the fitting procedure is presented in Appendix 2 of the Supplementary Information. To demonstrate the effectiveness of this approach and its implementation in the PAOFLOW package, we present results for four prototypical systems: GaAs, Si, Mg$_3$Sb$_2$ and CoSb$_3$.

### Computational details and implementation in PAOFLOW

The calculation of the Boltzmann transport with the modified RTA models is implemented in PAOFLOW and follow a standard algorithmic flow. PAOFLOW requires a few basic calculations performed with the Quantum ESPRESSO (QE) package^24,25^. The first (self-consistent) run generates a converged electronic density and Kohn–Sham (KS) potential on an appropriate Monkhorst and Pack (MP) **k**-point mesh. The second (non self-consistent) one evaluates eigenvalues and eigenfunctions on a larger MP mesh and often for an increased number of bands. After these preliminary steps PAOFLOW’s most fundamental procedure is the construction of accurate PAO Hamiltonians following the theory outlined in section “PAOFLOW”.

The density functional theory (DFT) calculations for Si were performed using a local density approximation (LDA). A kinetic energy cut-off of 18 Ry (180 Ry cut-off for the charge density) and a $12 \times 12 \times 12$ Monkhorst–Pack **k**-point mesh were used for the non self-consistent calculation. This was further increased to a $150 \times 150 \times 150$ grid using PAOFLOW’s Fourier interpolation method in order to accurately integrate transport tensors.

The DFT calculations for GaAs were performed using the generalized gradient approximation (GGA) in the parametrization of Perdew, Burke, and Ernzerhof (PBE). Projector augmented wavefunctions (PAW) pseudopotentials were used to treat the ion-electron interactions. A kinetic energy cut-off of 60 Ry (600 Ry cut-off for the charge density) and a $16 \times 16 \times 16$ Monkhorst–Pack **k**-point mesh were used. This was further increased to a $100 \times 100 \times 100$ grid using PAOFLOW (The convergence of electronic conductivity as a function of k-grid size is shown in Appendix 4 of the Supplementary Information).

The DFT calculations for ${\text {Mg}}_{3}{\text {Sb}}_{2}$ were performed using a GGA functional in the parametrization of PBE. PAW were used to treat the ion-electron interactions. A kinetic energy cut-off of 45 Ry (450 Ry cut-off for the charge density) and a $24 \times 24 \times 18$ Monkhorst–Pack **k**-point mesh were used. This was further increased to a $96 \times 96 \times 72$ grid using PAOFLOW.

The DFT calculations for ${\text {CoSb}}_{3}$ were performed using a LDA functionals. A kinetic energy cut-off of 45 Ry (450 Ry cut-off for the charge density) and a $10 \times 10 \times 10$ Monkhorst–Pack **k**-point mesh were used. This was further increased to a $100 \times 100 \times 100$ grid using PAOFLOW. The pseudopotentials for all the atomic species were obtained from pslibrary1.0.0^26^.

The experimental parameters used in the calculations of the relaxation times for different systems have been listed in Table 2. The energy, E is taken from the original DFT Hamiltonian processed by PAOFLOW. The calculations are done for user defined ranges of temperature T. An example workflow is discussed in Appendix 3 of the Supplementary Information. The relaxation time as a function of energy obtained using models have been compared to those obtained from first principles^27–29^ in Appendix 4 of the Supplementary Information. A reasonable qualitative agreement was observed with the additional benefit of low computational cost.

**Table 2:** Symbols and units of the parameters to be input in calculation of scattering models. The values for Si and GaAs are obtained from Ref.^18^, for ${\text {Mg}}_3{\text {Sb}}_{2}$ from Ref.^20^ and for ${\text {CoSb}}_{3}$ from Refs.^30,31^.

Symbol	Units	${\text {Mg}}_{3}{\text {Sb}}_{2}$	GaAs	Si	${\text {CoSb}}_{3}$
$\rho$	kg/m$^{3}$	$3.9 \times 10^{3}$	$5.3\times 10^{3}$	$2.3\times 10^{3}$	$7.8\times 10^{3}$
a	m	$8.7\times 10^{-10}$	$5.6\times 10^{-10}$	$5.4\times 10^{-10}$	$9.1\times 10^{-10}$
$\epsilon _{0}$	–	26.7	13.5	11.7	33.5
$\epsilon _{\infty }$	–	14.2	11.6	–	25.6
v	m/s	$2.7\times 10^{3}$	$5.2\times 10^{3}$	$6.6\times 10^{3}$	$3.3\times 10^{3}$
m$^{*}$	–	0.3	0.7	0.29	3
D$_{ac}$	eV	6.5	7	9.5	5
D$_{op}$	eV	–	–	$8\times 10^{10}$	$1 \times 10^{11}$
$\hbar \omega _{LO}$	eV	[0.0205,0.0248,0.031]	[0.03536]	–	[0.0264]
p	C/m$^{2}$	–	0.16	–	–

### GaAs

The scattering models were implemented on n-type GaAs for two different carrier concentrations, $3.5\times 10^{17}\;{\text {cm}}^{-3}$ and $7.7\times 10^{18}\;{\text {cm}}^{-3}$ and was used to calculate conductivities.

**Figure 4:**
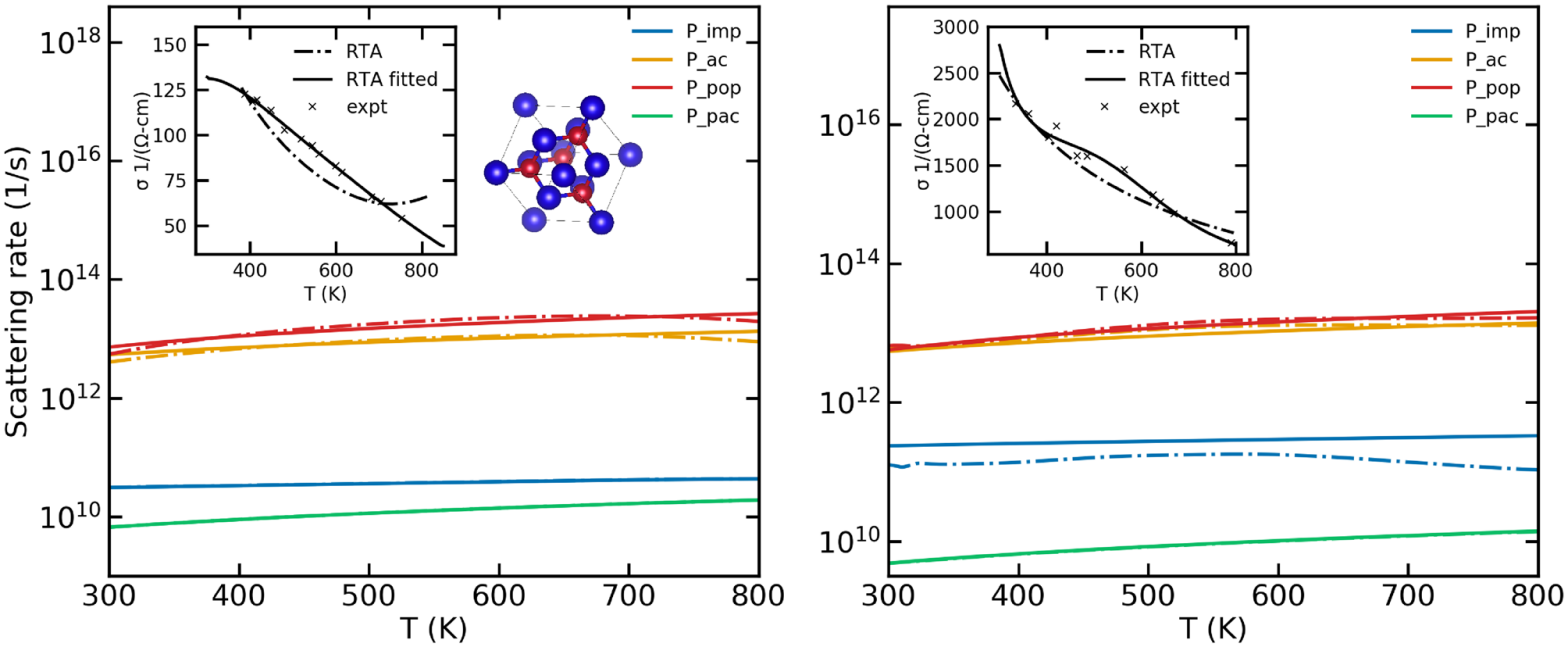
A comparison of the scattering rates in GaAs obtained using the original scattering models (labelled RTA) and those obtained using the fitting procedure (labelled RTA fitted) for samples of two different doping concentrations. The left panel corresponds to an n-type sample with a doping concentration of $3.5\times 10^{17}\,{\text {cm}}^{-3}$ while the right panel corresponds to an n-type doping of $7.7\times 10^{18}\,{\text {cm}}^{-3}$. The inset shows the electrical conductivity calculated using both the original and the fitted scattering rates.The experimental data have been obtained from Ref.^32^.

The scattering rates as a function of temperature are depicted by the solid lines in Fig. 4. The dominant scattering mechanisms were determined from Refs.^32^ and^33^. The calculated conductivities were then compared to the experimental values as shown in the inset of Fig. 4. The fitting procedure is performed as well and the scattering rates obtained as a result of the fitting procedure are shown by the dashed lines in Fig. 4. The fitting procedure produces negligible changes to the scattering rates, signifying that the original models themselves represent the scattering rates in GaAs well.

Reference^32^, from which the experimental data have been obtained, uses analysis of their Seebeck and Hall coefficient data and notes that the relative weight of polar scattering increases with increasing temperature, whereas the contribution of impurity scattering decreases with increasing temperature. This is confirmed by our results as well.

### Si

**Figure 5:**
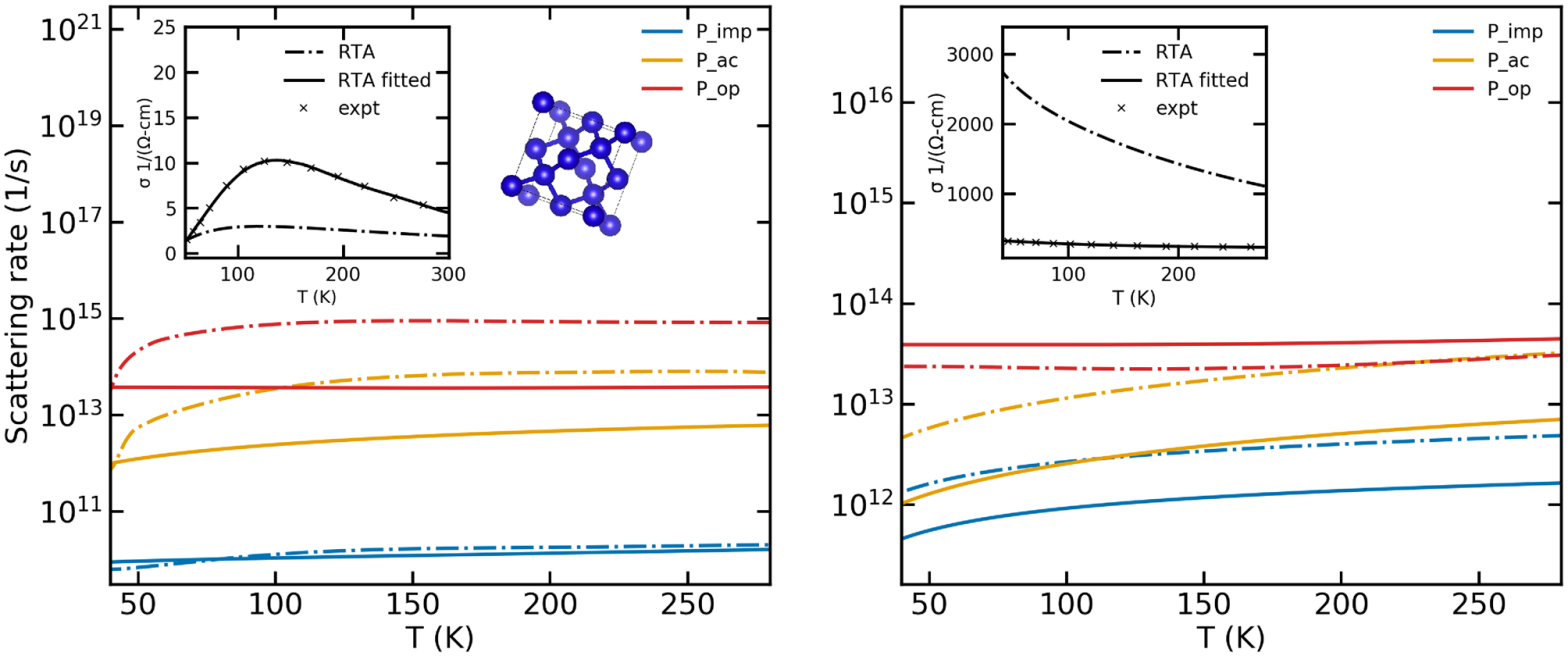
Conductivities and scattering rates for the different Si samples in a low temperature regime. The left panel shows the data for a sample with n-type doping with a carrier concentration of $2.8 \times 10^{16}$ cm$^{-3}$ while the right panel shows the data for a sample with n-type doping with a carrier concentration of $1.7 \times 10^{19}$ cm$^{-3}$. The inset shows the goodness of fit of theoretical electrical conductivity to experiments resulting from the fitting procedure as well as the electrical conductivity calculated using the original scattering models

Electronic conductivities were calculated for intrinsic Si, and n-type Si with doping concentrations of $2.8\times 10^{16}$ cm$^{-3}$ and $1.7\times 10^{19}$ cm$^{-3}$. Similar to GaAs, the solid lines in Fig. 5 represent the scattering rates calculated from the original models while the dashed lines represent the scattering rates obtained using the modified Mathiessen’s rule. It is clear from the results that the original scattering rates require significant modification for the calculated conductivities to match experiments.

Low temperature experimental data for transport properties in Si were obtained from Ref.^34^. There, the authors discuss the electrical conductivity of the heavily doped sample ($n =1.7 \times 10^{19}$ cm$^{-3}$) being weakly temperature dependent due to the large number of impurity atoms forming an impurity band. They state that in contrast, the weakly doped $n=2.8 \times 10^{16}$ cm$^{-3}$ exhibits an exponential behavior of electrical conductivity due to the freeze out of impurities at low temperatures. This behavior is fully captured by our models as well. In panel 1 of Fig. 5, for $n=2.8 \times 10^{16}$ cm$^{-3}$ the fitting procedure produces negligible change to the scattering rate due to impurity scattering in order to match experimental data. However, in panel 2 of Fig. 5, for $n=1.7 \times 10^{19}$ cm$^{-3}$ we see the contribution of impurities to the overall scattering rate is underestimated by the original models, but is rectified by our fitting procedure which significantly increases the scattering rate due to impurities. The dominant scattering mechanisms are the optical and acoustic phonons scattering whose values require significant correction by the fitting procedure.

### Mg$_3$Sb$_2$

**Figure 6:**
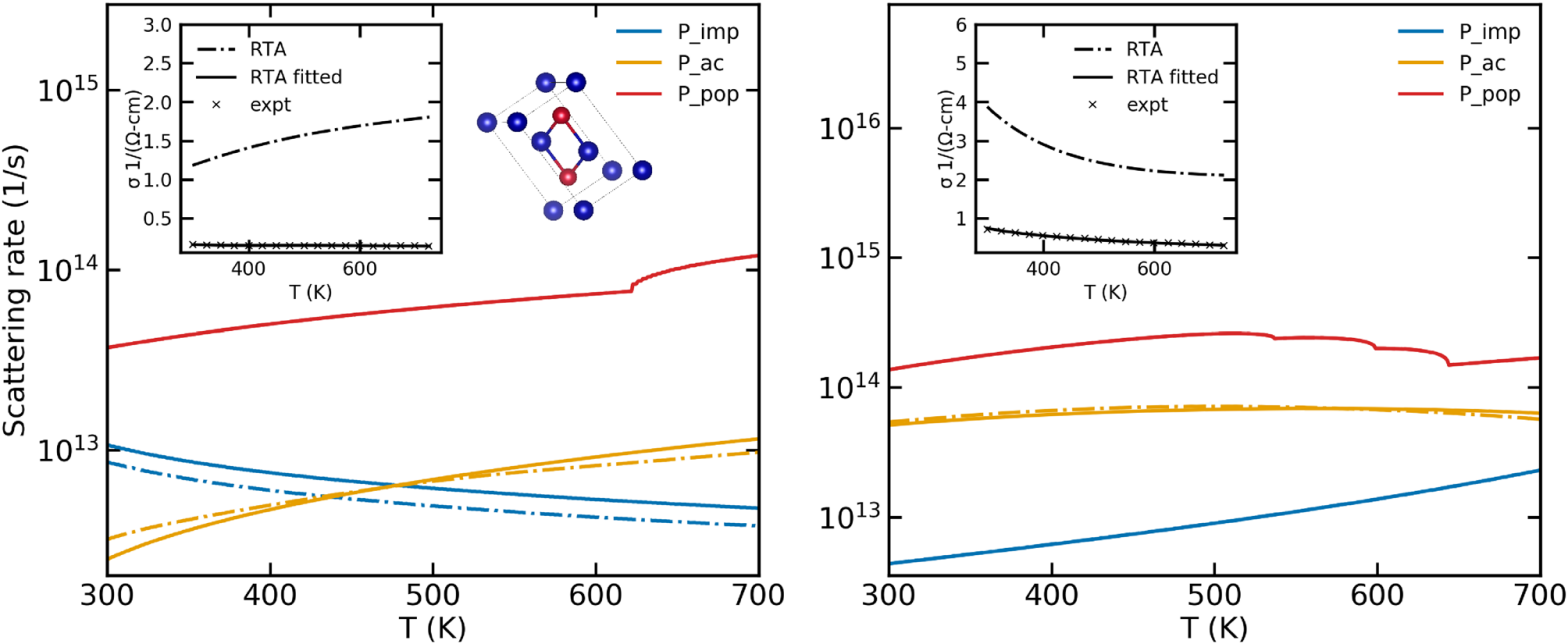
A comparison of the scattering rates in Mg$_3$Sb$_2$ obtained using the original scattering models (RTA) and those obtained using the fitting procedure (RTA$_{\text {fitted}}$). The left panel corresponds to an n-type sample with a doping concentration of $3.6\times 10^{18}\,{{\text {cm}}}^{-3}$ while the right panel corresponds to an n-type doping of $3.6\times 10^{19}\,{\text {cm}}^{-3}$. The inset shows the electrical conductivity calculated using the respective scattering rates.The experimental data have been obtained from Ref.^20^

${\text {Mg}}_{3}{\text {Sb}}_{2}$, a well studied thermoelectric material was chosen as a test system in order to verify our implementation of the various scattering models as well as the fitting procedure. The effect of scattering rates on transport properties in ${\text {Mg}}_{3}{\text {Sb}}_{2}$ and comparison to experiments have been extensively carried out in Ref.^20^. Their results show that transport properties calculated with scattering models are in good agreement with experiments. This is confirmed by our implementation of scattering models and the subsequent fitting procedure. As shown in Fig. 6, the fitting procedure produces negligible modifications to the original scattering models in order to match calculated electrical conductivity to experimental data.

### CoSb$_3$

**Figure 7:**
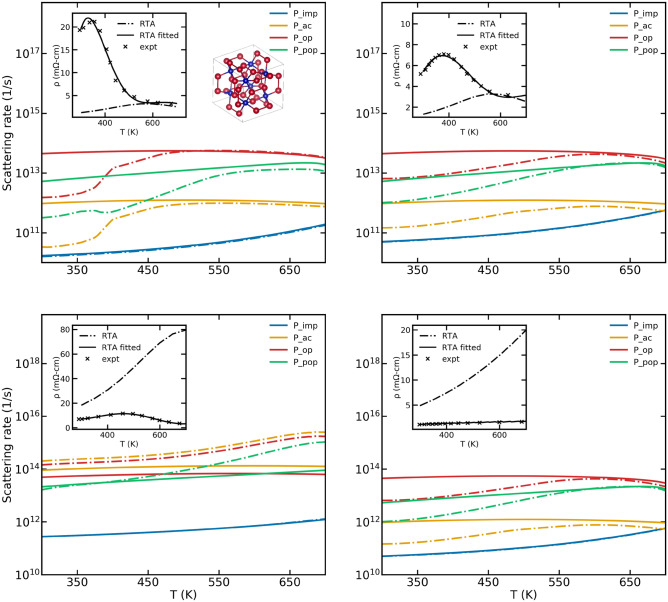
A comparison of the scattering rates in CoSb$_3$ obtained using the original scattering models (RTA) and those obtained using the fitting procedure (RTA$_{\text {fitted}}$) are shown. The top left panel corresponds to a p-type sample with a doping concentration of $1.2\times 10^{17}\,{\text {cm}}^{-3}$ while the top right panel corresponds to a p-type doping of $4.4\times 10^{17}\,{\text {cm}}^{-3}$. The bottom left panel corresponds to an n-type sample with a doping concentration of $152\times 10^{17}\,{\text {cm}}^{-3}$ while the bottom right panel corresponds to an n-type doping of $1380\times 10^{17}\,{\text {cm}}^{-3}$. The inset shows the electrical resistivity calculated using the both the original and fitted scattering rates. The experimental data have been obtained from Ref.^35^.

The conductivity for the p-type samples of ${\text {CoSb}}_{3}$ seem better represented by the scattering models than that of those for n-type samples. As seen in Fig. 7, the scattering rates for n-type sample require significant corrections at higher temperatures in order to reproduce experimental results.

CoSb$_3$, a well known thermoelectric, is studied over a wide range of temperatures. Caillat et al.^35^ analyzed their experimental mobility data for p-type samples and suggests that the dominant scattering mechanism, at least below 500 K is acoustic phonon scattering since the mobility followed a T$^{-3/2}$ behaviour. However, our models seem to suggest that the dominant scattering mechanism is optical phonon scattering for p-type samples and depending on the doping levels, maybe acoustic phonon scattering or optical phonon scattering for n-type samples. This inconsistency is also noted by Kajikawa^36^ who carried out analysis of p-type CoSb$_3$ within a two-valence and two-conduction band model.

## Conclusion

We have implemented relaxation time models that allow calculation of conductivities beyond the constant relaxation time approximations and are able to provide reasonable agreement to experimental conductivities in various systems. Moreover, unlike the CRTA, it allows for a quantitative and qualitative description of the scattering mechanisms themselves. We introduce an automated self consistent fitting procedure that allows one to see how various the base scattering models need to be tuned in order to reproduce experimental conductivities. This is highly advantageous in determining sample specific scattering properties which is beyond the scope of the base models. Future work includes the implementation of mobility using the BTE and therefore the extension of PAOFLOW as a tool to analyze experimental mobility data to extract scattering time trends.

## Supplementary Information

Supplementary Information.

## References

[1] Xu, B. & Verstraete, M. J. First principles explanation of the positive Seebeck coefficient of lithium. *Phys. Rev. Lett.* **112**, 196603 (2014). 10.1103/PhysRevLett.112.196603 24877957

[2] Zhou J (2018). Large thermoelectric power factor from crystal symmetry-protected non-bonding orbital in half-Heuslers. Nat. Commun..

[3] Sun P (2015). Large Seebeck effect by charge-mobility engineering. Nat. Commun..

[4] Zhou, J.-J. *et al.* Perturbo: A software package for ab initio electron-phonon interactions, charge transport and ultrafast dynamics. *Comput. Phys. Commun.* **264**, 107970 (2021).

[5] Poncé S, Margine ER, Verdi C, Giustino F (2016). EPW: Electron–phonon coupling, transport and superconducting properties using maximally localized Wannier functions. Comput. Phys. Commun..

[6] Shuai J (2017). Tuning the carrier scattering mechanism to effectively improve the thermoelectric properties. Energy Environ. Sci..

[7] Ricci, F. *et al.* An ab initio electronic transport database for inorganic materials . *Sci. Data* **4**, 1–13 (2017). 10.1038/sdata.2017.85 PMC5496472 28675382

[8] Buongiorno Nardelli, M. *et al.* Paoflow: A utility to construct and operate on ab initio hamiltonians from the projections of electronic wavefunctions on atomic orbital bases, including characterization of topological materials. *Comput. Mater. Sci.* **143**, 462 – 472 (2018).

[9] Cerasoli, F. T. *et al.* Advanced modeling of materials with PAOFLOW 2.0: New features and software design. *Comput. Mater. Sci.* **200**, 110828 (2021). PAOFLOW ver. 2.0 at https://github.com/marcobn/PAOFLOW

[10] Agapito, L. A., Ferretti, A., Calzolari, A., Curtarolo, S. & Buongiorno Nardelli, M. Effective and accurate representation of extended Bloch states on finite Hilbert spaces. *Phys. Rev. B* **88**, 165127 (2013).

[11] Agapito, L. A., Ismail-Beigi, S., Curtarolo, S., Fornari, M. & Buongiorno Nardelli, M. Accurate tight-binding Hamiltonian matrices from ab initio calculations. Minimal basis sets. *Phys. Rev. B* **93**, 035104-9 (2016).

[12] Agapito LA (2016). Accurate tight-binding Hamiltonians for two-dimensional and layered materials. Phys. Rev. B.

[13] D’Amico, P. *et al.* Accurate ab initio tight-binding hamiltonians: Effective tools for electronic transport and optical spectroscopy from first principles. *Phys. Rev. B* **94**, 165166. 10.1103/PhysRevB.94.165166 (2016).

[14] Parravicini, G. P. & Grosso, G. *Solid State Physics*, 1st edn (Academic Press, 2000).

[15] Singh, D. J. Theoretical and computational approaches for identifying and optimizing novel thermoelectric materials. In Tritt, T. M. (ed.) *Recent Trends in Thermoelectric Materials Research II*, *Semiconductors and Semimetals*, Vol. 70, 125 – 177 (Elsevier, 2001).

[16] Madsen, G. K. & Singh, D. J. Boltztrap. A code for calculating band-structure dependent quantities. *Comput. Phys. Commun.* **175**, 67 – 71 (2006).

[17] Mecholsky, N. A., Resca, L., Pegg, I. L. & Fornari, M. Theory of band warping and its effects on thermoelectronic transport properties. *Phys. Rev. B* **89**, 155131 (2014).

[18] Jacoboni, C. *Theory of Electron Transport in Semiconductors: A Pathway from Elementary Physics to Nonequilibrium Green Functions*, Vol. 165 (Springer Science & Business Media, 2010).

[19] Madsen, G. K. & Singh, D. J. BoltzTraP. A code for calculating band-structure dependent quantities. *Computer Phys. Commun.* **175**, 67–71 (2006).

[20] Farris, R., Maccioni, M. B., Filippetti, A. & Fiorentini, V. Theory of thermoelectricity in Mg3Sb2 with an energy-and temperature-dependent relaxation time. *J. Phys. Condens. Matter* **31**, 065702 (2018). 10.1088/1361-648X/aaf364 30524117

[21] Ridley B (1998). Polar-optical-phonon and electron–electron scattering in large-bandgap semiconductors. J. Phys. Condens. Matter.

[22] Long D, Myers J (1959). Ionized-impurity scattering mobility of electrons in silicon. Phys. Rev..

[23] Nocedal, J. & Wright, S. J. Sequential quadratic programming. *Numer. Optim.*, 529–562 (2006).

[24] Giannozzi, P. *et al.* QUANTUM ESPRESSO: A modular and open-source software project for quantum simulations of materials. *J. Phys. Condens. Matter* **21**, 395502 (2009). 10.1088/0953-8984/21/39/395502 21832390

[25] Giannozzi, P. *et al.* Advanced capabilities for materials modelling with quantum espresso. *J. Phys. Condens. Matter* **29**, 465901 (2017). QE ver. 6.4 at https://www.quantum-espresso.org 10.1088/1361-648X/aa8f79 29064822

[26] Dal Corso A (2014). Pseudopotentials periodic table: From h to pu. Comput. Mater. Sci..

[27] Ganose, A. M. *et al.* Efficient calculation of carrier scattering rates from first principles. *Nat. Commun.* **12**, 1–9 (2021). 10.1038/s41467-021-22440-5 PMC8044096 33850113

[28] Poncé, S. *et al.* Towards predictive many-body calculations of phonon-limited carrier mobilities in semiconductors. *Phys. Rev. B* **97**, 121201 (2018).

[29] Zhou, J.-J. & Bernardi, M. Ab initio electron mobility and polar phonon scattering in GaAs. *Phys. Rev. B* **94**, 201201 (2016).

[30] Tang Y (2015). Convergence of multi-valley bands as the electronic origin of high thermoelectric performance in CoSb 3 skutterudites. Nat. Mater..

[31] Arushanov E, Fess K, Kaefer W, Kloc C, Bucher E (1997). Transport properties of lightly doped CoSb 3 single crystals. Phys. Rev. B.

[32] Amith A, Kudman I, Steigmeier E (1965). Electron and phonon scattering in GaAs at high temperatures. Phys. Rev..

[33] Lee H, Basinski J, Juravel L, Woolley J (1979). Electrical transport and band structure of GaAs. Can. J. Phys..

[34] Weber L, Gmelin E (1991). Transport properties of silicon. Appl. Phys. A.

[35] Caillat T, Borshchevsky A, Fleurial J-P (1996). Properties of single crystalline semiconducting CoSb3. J. Appl. Phys..

[36] Kajikawa, Y. Analysis of high-temperature thermoelectric properties of p-type CoSb3 within a two-valence-band and two-conduction-band model. *J. Appl. Phys.* **115**, 203716 (2014).

[37] Giustino, F. Electron–phonon interactions from first principles. *Rev. Mod. Phys.* **89**, 015003 (2017).

