# Supplementary material for: Relaxation time approximations in PAOFLOW 2.0
Source: Sci Rep. 2022 Mar 23;12:4993. doi: 10.1038/s41598-022-08931-5 (PMC8943075; doi:10.1038/s41598-022-08931-5)
Supplement: Supplementary file 1 — Supplementary Information. [file 41598_2022_8931_MOESM1_ESM.pdf]

Supplementary Information : Relaxation time approximations in PAOFLOW 2.0

Anooja Jayaraj[1,*], Ilaria Siloi[2], Marco Fornari[3], and Marco Buongiorno Nardelli[1,4]

[1]Department of Physics, University of North Texas, Denton, TX 76203, USA
[2]Department of Physics and Astronomy, University of Southern California, Los Angeles, CA 90007, USA
[3]Department of Physics and Science of Advanced Materials Program, Central Michigan University, Mt.Pleasant, MI 48859
[4]Santa Fe Institute, Santa Fe, NM 87501, USA
[*]AnoojaJayaraj@my.unt.edu

1 Appendix 1

Formulas of the functions entering the definition of the optical phonon scattering time:

$$A(E,T,\omega_{op}) = n(\omega_{op}+1)\frac{f_0(E+\hbar\omega_{op})}{f_0(E)}\left[(2E+\hbar\omega_{op})sinh^{-1}\left(\frac{E}{\hbar\omega_{op}}\right)^{\frac{1}{2}} - [E(E+\hbar\omega_{op})]^{\frac{1}{2}}\right] \tag{1}$$

$$B(E,T,\omega_{op}) = \theta(E-\hbar\omega_{op})n(\omega_{op})\frac{f_0(E-\hbar\omega_{op})}{f_0(E)}\left[(2E-\hbar\omega_{op})cosh^{-1}\left(\frac{E}{\hbar\omega_{op}}\right)^{\frac{1}{2}} - [E(E-\hbar\omega_{op})]^{\frac{1}{2}}\right] \tag{2}$$

$$C(E,T,\omega_{op}) = 2E\left[n(\omega_{op}+1)\frac{f_0(E+\hbar\omega_{op})}{f_0(E)}sinh^{-1}\left(\frac{E}{\hbar\omega_{op}}\right)^{\frac{1}{2}} + \theta(E-\hbar\omega_{op})n(\omega_{op})\frac{f_0(E-\hbar\omega_{op})}{f_0(E)}cosh^{-1}\left(\frac{E}{\hbar\omega_{op}}\right)^{\frac{1}{2}}\right] \tag{3}$$

$$Z(\omega_{op}) = \frac{2}{W_0(\hbar\omega_{op})^{\frac{1}{2}}},\, W_0(\omega_{op}) = \frac{e^2\sqrt{2m^*\omega_{op}}\varepsilon^{-1}}{4\pi\hbar^{\frac{3}{2}}} \tag{4}$$

2 Appendix 2

We implemented a fitting procedure as follows. We extract experimental data for the electronic conductivity (or resistivity) as a function of temperature for the system of interest. Since the experimental data points may be few and far apart, this data is interpolated using a polynomial fit of a degree that best fits the available data. This will allow for a smooth fitting procedure without nonphysical discontinuities in the final scattering times. The fitting proceeds to minimize the distance $f(T)$ between the experimental conductivity curve and the calculated conductivity curve by varying the fitting functions (a_{im}, a_{ac}, a_{op}...), where it is assumed that the functions vary with temperature:

$$f(T) = \sum_{i=1}^{N}\frac{(\sigma^i_{exp}(T)-\sigma^i_{pao}(T))^2}{N}, \tag{5}$$

where σ_{exp} and σ_{pao} are the experimental and the theoretical values of conductivity respectively, with N being total number of data points taken into consideration. Since the conductivity curves often span a wide range of temperatures and show different behaviors in different ranges, we introduce a moving overlapping bin fitting procedure to capture these changes and obtain smooth fits. This is done by splitting both the experimental and calculated data into overlapping bins with at least f data points, f being the number of fitting parameters. Therefore the bins for the experimental conductivities with four fitting

parameters would be
$[\ \sigma_{exp}(T_1), \sigma_{exp}(T_2), \sigma_{exp}(T_3), \sigma_{exp}(T_4)]$, $[\ \sigma_{exp}(T_2), \sigma_{exp}(T_3), \sigma_{exp}(T_4), \sigma_{exp}(T_5)]$ and so on, covering the entire temperature range. The calculated conductivities are split into similar bins and the distance between the curves represented by the calculated conductivity bin and the experimental conductivity bin is minimized using the following steps.
Set the initial guess for the fitting parameters (a_{imp}, a_{ac}, a_{op}...) of the first bin to ones so that the starting point for the of the fitting procedure coincides with the familiar Matthiessen's rule (Eq. 14). The total scattering time as a function of $E(\mathbf{k},T)$ obtained from Eq. 24 is plugged into Eq. 6 in order to calculate the electrical conductivity. The fitting parameters are allowed to vary until the distance between the first bin of the experimental curve and the first bin of the calculated curve is minimized. We will refer to these set of fitting parameters as the converged fitting parameters and the relaxation times calculated using these converged fitting parameters as converged relaxation times.
This minimization procedure is carried out for every bin. In order to speed up the fitting procedure, the converged fitting parameters of the previous bin is taken to be the initial guess for the fitting parameters of every subsequent bin. This also removes any nonphysical jumps in relaxation times between the bins.
The converged relaxation times obtained for every temperature in every bin is combined. Since the bins overlap in temperature, every temperature will have multiple relaxation times as well. These are averaged to obtain one converged relaxation time per temperature to obtain the final relaxation time vs temperature plot.

3 Appendix 3

Listing of the main.py from example 10 of the PAOFLOW package where we construct the workflow to reproduce the results of GaAs with a doping concentration of $3.5 \times 10^{17}\ cm^{-3}$ described in Section 4.2. For a complete discussion of method and attributes of the PAOFLOW class see[1]. Once the interpolated Hamiltonian is built and the gradient and momenta are computed, various transport properties can be calculated for any system. Since the systems are typically doped, **doping_conc** argument is passed to the *doping* routine which computes the chemical potential required to fix the doping concentration for various temperatures. The energy and temperature dependent scattering models are defined with the `TauModel` class. The built in models include acoustic, optical, polar acoustic, polar optical and ionized impurity scattering. These built-in models only require the specification of empirical constants. The empirical constants required for any selected built-in models are passed into a python dictionary as the **tau_dict** argument of the `transport` routine. PAOFLOW also allows the user to define scattering models that can be passed directly to the **scattering_channels** argument as demonstrated by the **acoustic_model** function. The variable **channels** then uses 1 user-defined and 3 built-in scattering model. The listing below then calculates the transport properties for each user defined temperature and calculated chemical potential using the RTA for user defined scattering models. It outputs a file containing the electronic conductivities (σ) that were used to compare to experiments. The code for the fitting procedure described in Appendix 2 is shown in the Listing 3 and 4. This reproduces the fitted conductivity curve shown in the inset of Figure 4 and is used to calculate the so called fitted scattering rates.

Listing 1: `main.py` **- Transport coefficients for GaAs - Base models**

```
import numpy as np
from  PAOFLOW import  PAOFLOW
from  PAOFLOW.defs.TauModel import TauModel

def main():

   PAOFLOW =  PAOFLOW. PAOFLOW(savedir='GaAs.save', smearing=None, npool=1, verbose=
       True)
  arrays,attr =  PAOFLOW.data_controller.data_dicts()
   PAOFLOW.read_atomic_proj_QE()
   PAOFLOW.projectability()
   PAOFLOW.pao_hamiltonian()
   PAOFLOW.interpolated_hamiltonian(nfft1=100, nfft2=100, nfft3=100)
   PAOFLOW.pao_eigh()
   PAOFLOW.gradient_and_momenta()

  doping = -3.5e17
   PAOFLOW.doping(tmin=380, tmax=812, nt=28, emin=-36, emax=2, ne=5000, doping_conc=
       doping)

  me = 9.10938e-31 # Electron Mass
  ev2j = 1.60217662e-19 # Electron Charge

  def acoustic_model ( temp, eigs, params ):

    from scipy.constants import hbar
    temp *= ev2j
    E = eigs * ev2j # Eigenvalues in J
    v = 5.2e3 # Velocity in m/s
    rho = 5.31e3 # Mass density kg/m^3
    ms = .7 * me #effective mass tensor in kg
    D_ac = 7 * ev2j # Acoustic deformation potential in J
    return (2*ms)**1.5*(D_ac**2)*np.sqrt(E)*temp/(2*np.pi*rho*(hbar**2*v)**2)

  acoustic_tau = TauModel(function=acoustic_model)

  fname = 'doping_n%s.dat'%np.abs(doping)
  temp = np.loadtxt('output/%s'%fname, usecols=(0,))
  mu = np.loadtxt('output/%s'%fname, usecols=(1,))
```

Listing 2: `main.py - continuation` **- Transport coefficients for GaAs - Base models**

```
  channels = [acoustic_tau, 'polar_optical', 'impurity', 'polar_acoustic']

  tau_params = {'doping_conc':-3.5e17, 'D_ac':7., 'rho':5.31e3,
                'a':5.653e-10, 'nI':3.5e17, 'eps_inf':11.6, 'eps_0':13.5,
                'v':5.2e3, 'Zi':1, 'hwlo':[0.03536], 'D_op':3e10, 'Zf':6,
                'piezo':0.16, 'ms':0.7, 'Ef':0.0}

  rho = []
  for t,m in zip(temp,mu):
    if  PAOFLOW.rank == 0:
      print('\nTemp, Mu: %f, %f'%(t,m))

     PAOFLOW.transport(tmin=t, tmax=t, nt=1, emin=m, emax=m, ne=1, scattering_channels
         =channels, tau_dict=tau_params, save_tensors=True, write_to_file=False)

    sigma = np.sum([sig for sig in np.diag(arrays['sigma'][:,:,0])])/3
    rho.append(1e2/sigma)

  if  PAOFLOW.rank == 0:
    with open('output/rho_rta_n3.5e17.dat' ,'w') as rho_file:
      for i,t in enumerate(temp):
        rho_file.write('%8.2f %9.5e\n'%(t,rho[i]))

   PAOFLOW.finish_execution()

if __name__== '__main__':
  main()
```

Listing 3: `main.py` **- Transport coefficients for GaAs - Fitted models**

```
from  PAOFLOW import  PAOFLOW
from  PAOFLOW.defs.TauModel import TauModel
import numpy as np
import scipy.optimize
import sys

def main():

   PAOFLOW =  PAOFLOW. PAOFLOW(savedir='GaAs.save', smearing=None, npool=1, verbose=
       True)
  arrays,attr =  PAOFLOW.data_controller.data_dicts()
   PAOFLOW.read_atomic_proj_QE()
   PAOFLOW.projectability()
   PAOFLOW.pao_hamiltonian()
   PAOFLOW.interpolated_hamiltonian(nfft1=100, nfft2=100, nfft3=100)
   PAOFLOW.pao_eigh()
   PAOFLOW.gradient_and_momenta()

  def get_curve_eqn(x_data,y_data,x,degree):
     a  = np.polyfit(x_data,y_data,degree)
     a = np.array(a[::-1])
     curve_eqn = sum(a[j]*np.power(x,j) for j in range(len(a)))
     return curve_eqn

  def data_bin(unbinned_data,bin_size):
     binned_data = []
     for i in range(0,len(unbinned_data)-bin_size+1):
        binned_data.append(unbinned_data[i:i+bin_size])
     return np.array(binned_data)

  def cost_func(par,temp,mu,y_expt):
     y_calc = cost_func_driver(temp,mu,par)
     y_err = np.sum((y_calc-y_expt)**2)/len(y_expt)
     return y_err

  def cost_func_driver(temp,mu,par):
     pao_rho_list = []
     for t,m in zip(temp,mu):
         PAOFLOW.transport(tmin = t,tmax = t,nt = 1,emin=m, emax=m,ne = 1,
             scattering_channels=['polar_optical','impurity','polar_acoustic','acoustic
             '],tau_dict={'doping_conc':-3.5e17,'Ef':m,'D_ac':7.,'rho':5.31e3,'a':5.653
             e-10,'nI':3.5e17,'eps_inf':11.6,'eps_0':13.5,'v':5.2e3,'Zi':1,'hwlo'
             :[0.03536],'D_op':3e10,'Zf':6,'piezo':0.16,'ms':0.291},a_imp=par[0],a_ac=
             par[1],a_pop=par[2],a_pac=par[3],write_to_file=False)
        pao_sigma = (arrays['sigma'][0,0]+arrays['sigma'][1,1]+arrays['sigma'][2,2])/3
        pao_rho = (1e2/pao_sigma) #convert to match units of expt data, ohm-cm
        pao_rho_list.append(pao_rho)
     return np.array(pao_rho_list)


  def cost_func_optimize(par_guess,bounds,temp,mu,y_expt):
     par_optimal = scipy.optimize.minimize(cost_func,par_guess,args=(temp,mu,y_expt),
         method='SLSQP',bounds=bounds,tol=0.0009)
     return par_optimal.x
```

Listing 4: `main.py - continuation` **- Transport coefficients for GaAs - Fitted models**

```
    x_expt = np.loadtxt('expt_data/amith_n3.5e17',usecols=(0,))
    y_expt = np.loadtxt('expt_data/amith_n3.5e17',usecols=(1,))
    temp = np.loadtxt('EvT_n3.5e17/dope_TvsE_-3.5e+17.dat',usecols=(0,))
    mu = np.loadtxt('EvT_n3.5e17/dope_TvsE_-3.5e+17.dat',usecols=(1,))
    y_expt_curve=[]

    for t in temp:
        y_expt_curve.append(get_curve_eqn(x_expt,y_expt,t,4))

    binned_temp = data_bin(temp,5)
    binned_mu = data_bin(mu,5)
    binned_y_expt = data_bin(y_expt_curve,5)

    for bin_no in range(len(binned_temp)):
        optimized_rho = open('optimized_rho_n3.5e17_bin_no_%s.dat'%bin_no,'w')
        optimized_S = open('optimized_S_n3.5e17_bin_no_%s.dat' %bin_no,'w')
        optimized_kappa = open('optimized_kappa_n3.5e17_bin_no_%s.dat' %bin_no,'w')
        if bin_no in range(1):
            par_guess = [1., 1., 1.,1.]
            bounds=((1e-9,100),(1e-9,100),(1e-9,100),(1e-9,100))
        else:
            par_guess = par_optimized
            bounds=((0.5*par_guess[0],1.5*par_guess[0]),(0.5*par_guess[1],1.5*par_guess
                [1]),(0.5*par_guess[2],1.5*par_guess[2]),(0.5*par_guess[3],1.5*par_guess
                [3]))
        par_optimized = cost_func_optimize(par_guess,bounds,binned_temp[bin_no,:],
            binned_mu[bin_no,:],binned_y_expt[bin_no,:])

        for t,m in zip(binned_temp[bin_no,:],binned_mu[bin_no,:]):
             PAOFLOW.transport(tmin = t,tmax = t,nt = 1,emin=m, emax=m,ne = 1,
                 scattering_channels=['polar_optical','impurity','polar_acoustic','
                 acoustic'],tau_dict={'doping_conc':-3.5e17,'Ef':m,'D_ac':7.,'rho':5.31e3
                 ,'a':5.653e-10,'nI':3.5e17,'eps_inf':11.6,'eps_0':13.5,'v':5.2e3,'Zi':1,
                 'hwlo':[0.03536],'D_op':3e10,'Zf':6,'piezo':0.16,'ms':0.291},a_imp=
                 par_optimized[0],a_ac=par_optimized[1],a_pop=par_optimized[2],a_pac=
                 par_optimized[3],write_to_file=False)
            pao_sigma = (arrays['sigma'][0,0]+arrays['sigma'][1,1]+arrays['sigma'][2,2])
                /3
            pao_rho = (1e2/pao_sigma) #convert to match expt, ohm-cm
            pao_S = (arrays['S'][0,0]+arrays['S'][1,1]+arrays['S'][2,2])/3
            pao_kappa = (arrays['kappa'][0,0]+arrays['kappa'][1,1]+arrays['kappa'][2,2])
                /3
            optimized_S.write('%8.2f␣%9.5e\n'%(t,pao_S))
            optimized_kappa.write('%8.2f␣%9.5e\n'%(t,pao_kappa))
            optimized_rho.write('%8.2f␣%.4f␣%.3f␣%.3f␣%.3f␣%.3f␣%9.5e\n'%(t,m,
                par_optimized[0],par_optimized[1],par_optimized[2],par_optimized[3],
                pao_rho))

        optimized_rho.close()
        optimized_S.close()
        optimized_kappa.close()

     PAOFLOW.finish_execution()

if __name__== '__main__':
    main()
```

4 Appendix 4

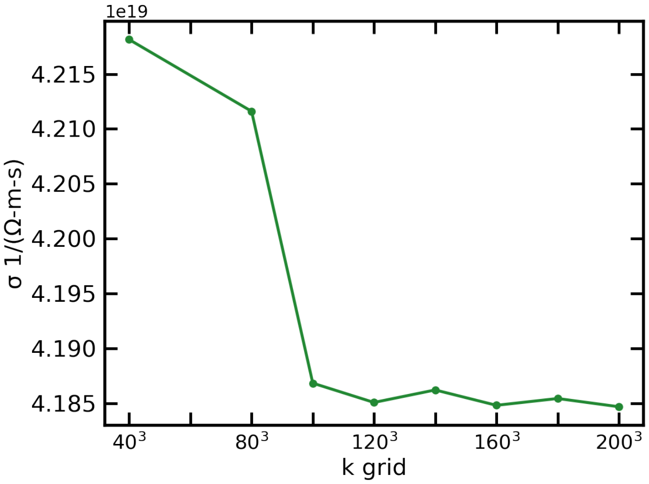

Supplementary Information Figure 1. The dependence of the electronic conductivity vs. k-grid density for GaAs. The analysis indicates that transport calculations have converged for 100^3 grid points.

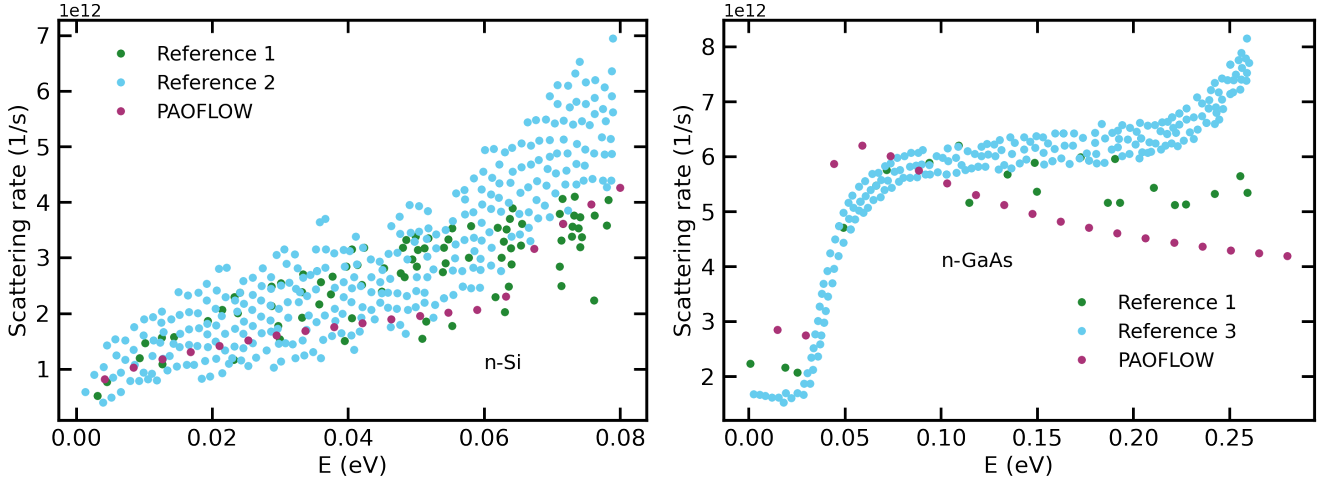

Supplementary Information Figure 2. A comparison of the scattering rates as a function of energy generated using models in PAOFLOW with first-principles scattering rates from Reference 1[2] and Reference 2[3] for n-type Si and Reference 3[4] for n-type GaAs. The parameters for scattering rates generated using PAOFLOW have been obtained from Supplementary Information of Reference 1[2]. At a qualitative level and for low-energy electrons, the comparison is acceptable, even more so if consideration related to the computational cost could be also included.

References

1. Cerasoli, F. T. *et al.* Advanced modeling of materials with paoflow 2.0: New features and software design. *Comput. Mater. Sci.* **200**, 110828 (2021).
2. Ganose, A. M. *et al.* Efficient calculation of carrier scattering rates from first principles. *Nat. communications* **12**, 1–9 (2021).
3. Poncé, S., Margine, E. R. & Giustino, F. Towards predictive many-body calculations of phonon-limited carrier mobilities in semiconductors. *Phys. Rev. B* **97**, 121201 (2018).
4. Zhou, J.-J. & Bernardi, M. Ab initio electron mobility and polar phonon scattering in gaas. *Phys. Rev. B* **94**, 201201 (2016).
